# Environmental and economic effectiveness of the Kyoto Protocol

**DOI:** 10.1371/journal.pone.0236299

**Published:** 2020-07-21

**Authors:** Yoomi Kim, Katsuya Tanaka, Shunji Matsuoka

**Affiliations:** 1 Department of Public Administration, Ewha Womans University, Seoul, Republic of Korea; 2 Research Centre for Sustainability and Environment, Shiga University, Shiga, Japan; 3 Graduate School of Asia-Pacific Studies (GSAPS), Waseda University, Tokyo, Japan; The Bucharest University of Economic Studies, ROMANIA

## Abstract

This study investigates the environmental and economic impacts of the Kyoto Protocol on Annex I parties through an impact assessment by combining the propensity score matching and the difference-in-difference methods. We establish a country-level panel data set including CO_2_ emissions, gross domestic product (GDP), and other socioeconomic data for 1997–2008 and 2005–2008. Based on the impact evaluation, we conduct the simulation predicting the impacts of the Protocol to capture the differences of marginal damage cost of carbon emissions between the actual and counterfactual situations. The results suggest that participating as an Annex I party has a significant positive impact on CO_2_ emission reductions, but a negative impact on the GDP of the participants in the long run. The predicted marginal benefit of the Protocol based on the marginal damage cost of carbon emissions shows that the marginal benefit of emission reductions mitigates a limited portion of the GDP loss. Future global climate change frameworks should focus on balancing the impact on economic and environmental performance in order to ensure sustainable development, especially for developing countries that have low capacity to mitigate emissions.

## Introduction

The intensification of transboundary environmental issues in the past half century has underscored the need to establish effective international instruments [1, 2]. International environmental agreements (IEA) help enhance transnational cooperation, such that global environmental degradation can be addressed [1]. States can commit to protect the environment by being a part of one or many IEAs [3].

The rise in the number of IEAs has led to a corresponding increase in the number of studies investigating and evaluating their effectiveness. Scholars have conducted quantitative analyses by applying diverse methodologies and establishing data sets to estimate the impact of IEAs. However, the results obtained in previous studies remain controversial. Proponents insist that an IEA has a significantly positive impact on improving environmental quality [4, 5], while opponents consider it an empty promise that involves large expenses for implementation [6–9]. The endemic nature of international policy—for example, many actors, different socioeconomic conditions among parties, analysis, and data sets on this topic—has become limited.

The Kyoto Protocol (hereafter, “Protocol”) is a highly influential IEA affiliated with the United Nations Framework Convention on Climate Change (UNFCCC) regime. This Protocol aims to mitigate greenhouse gas (GHG) emissions. It was adopted in 1997 in Kyoto and came into force in 2005. As of 2016, there are 192 parties to this protocol. The parties consist of 191 states and the European Union (EU) [10]. In 2016, the Paris Agreement was also adopted under the UNFCCC regime to prepare for the next step in addressing climate change [11].

According to the main principle of the 1992 UNFCCC, called “common but differentiated responsibilities and respective capabilities,” the Protocol considers differences in emissions, wealth, and capacity for change when allocating obligations toward emission reductions among parties [12]. Under Annex I of the Protocol, the principal sources of GHG emissions are listed. The list comprises Organization for Economic Cooperation and Development member countries and countries with economies in transition, at the relevant time. Accordingly, a heavier burden of internationally binding targets for emission reductions were imposed on Annex I parties. Moreover, this Protocol introduces three market-based mechanisms—International Emissions Trading, the Clean Development Mechanism (CDM), and Joint Implementation—to help countries successfully achieve their emission reductions targets [13, 14]. These flexible market mechanisms support Annex I parties in meeting their obligations toward reductions in a more cost-effective manner [15].

Previous studies have mostly analyzed the impacts of IEAs in terms of environmental aspects alone. While several studies indicate that carbon dioxide (CO_2_) emissions have decreased under the Protocol [8, 4, 5, 16–18], the environmental impact of the Protocol is still being disputed. Previous studies on the impact of the Protocol offer mixed results; most have failed to distinguish its impacts in terms of CO_2_ emission reductions from those of other socioeconomic effects. A suitable model is therefore needed to investigate the impact of the Protocol alone. For example, studies by Aakvik and Tjøtta [19], Kim et al. [20], and Vollenweider [21] demonstrate that advanced microeconometric approaches can estimate the impact of the Protocol on emissions reductions while discarding the effects of other factors on emissions. It is, thus, relevant to carry out an impact assessment in order to investigate the Protocol’s effectiveness in improving the environmental quality of Annex I countries.

Another important aspect of analyzing the Protocol from the perspective of sustainable development is its impact on economic performance, since various kinds of economic activity produce GHGs. Annex I parties with binding obligations for emission reductions may suffer a degree of economic decline because of technical difficulties and economic constraints [22]. Research by Nordhaus and Boyer [9] find that the policy is highly cost-ineffective. Some insist that industrialized countries suffer economic losses because of carbon abatement; however, as the Protocol has tried to reduce the cost of emission reductions by adopting market-based mechanisms, it is expected to achieve its aim of reducing emissions more efficiently by mitigating the associated costs [7, 23, 24].

For example, the CDM, which is introduced in Article 12 of the Protocol, may be a contributing factor. Under the CDM, Annex I parties can achieve a part of their emission reductions obligations of the Protocol by purchasing Certified Emission Reductions (CER) resulting from CDM projects in developing countries [10, 25]. In this case, buying CER can easily substitute their commitment toward meeting obligations within their own country, as comparatively lower capacity is effective in mitigating emissions in developing countries.

To summarize, it is difficult to grasp their net impact considering that various external factors, such as the socioeconomic conditions unique to each country, can influence the outcomes of the Protocol in the respective countries. Furthermore, although some previous studies have considered the economic consequences of the Protocol, none have analyzed its environmental *and* economic impacts under the same analytical framework. It is crucial to evaluate both the environmental and the economic impact to establish the most effective international environmental policies for achieving sustainable development.

Given this context, this study is the first to empirically investigate both the environmental and economic impacts of the Protocol on Annex I parties using the same microeconometric modeling approach. First, it investigates the Protocol’s environmental impact on CO_2_ emission reductions, particularly the contribution of Annex I parties toward these. Second, it estimates the net differences in the economic performance of each country before and after agreeing to participate in the Protocol as an Annex 1 party. Combining the propensity score matching (PSM) and difference-in-difference (DID) methods, this study conducts an impact assessment to accomplish its aim. This approach helps control for any unobserved internal and external effects. Thus, the environmental and economic impacts of the non-Annex I and the Annex I countries can be compared accurately. This process facilitates a deeper understanding of the impact of the Protocol.

## Materials and methods

### Matched DID model

This study carries out an impact assessment by combining the PSM and DID methods. It is possible to obtain unbiased and consistent estimates that control for both the selection bias and the problem of unobserved heterogeneity by using this model [26]. As this technique helps evaluate the impact of a certain treatment or program, it is gaining attention in various fields such as medical science, economics, and political science [27–29]. Nevertheless, it has seldom been applied to test the effectiveness of IEAs, with few exceptions. Analyses by Aakvik and Tjøtta [19], Grunewald & Martinez-Zarzoso [17], Kim et al. [20, 30, 31], and Vollenweider [21], for instance, have deployed the method.

The empirical procedure is as follows: First, the PSM method is applied to construct a statistical comparison group to measure the probability of participating in a program with observed characteristics, since unobserved macroeconomic changes or unknown socioeconomic characteristics between participants and non-participants could cause selection bias [26]. The propensity score, $\hat{P}$, can be estimated based on observed characteristics of the research objects *X*: $\hat{P}(X|T=1)=\hat{P}(X)$. The matched observations derived by the PSM method can be used for the matched DID.

The variables, *X*, represent the observed characteristics of Annex I and non-Annex I parties in this study. Each country’s gross domestic product (GDP), population, CO_2_ levels, and factors of production—capital, labor, and human capital—are included in the calculation of the propensity score. These variables are deemed to reflect the overall socioeconomic and environmental conditions in each country.

This study applies the DID matching algorithm to match participants to non-participants. To match participants with non-participants based on the propensity score, different matching algorithms can be used: nearest-neighbor matching, caliper or radius matching, stratification or interval matching, kernel and local linear matching, and DID matching. It uses country-level panel data that consists of observations in both the treatment and control group for time periods before and after the program. Thus, the DID matching algorithm can match the participant (treated) and non-participant (control) on the pre-program status, *X*. This matching algorithm assumes that the unobserved factors affecting participation are constant over time. Thus, the weights calculated by the propensity score for the base year are applied to the matched non-participants [26]. Country-level panel data on both Annex I and non-Annex I parties are available across the two time periods (i.e., before and after Protocol participation) to control for the selection of unobserved characteristics:

$${DD}_{i}=(Y_{i2}^{T}-Y_{i1}^{T})-\sum_{j\in C}\omega (i,j)(Y_{i2}^{C}-Y_{i1}^{C})$$ (1)

where $Y_{i}^{T}$ is unintended environmental and economic impacts of the Protocol on Annex I countries, and $Y_{i}^{C}$ is that of non-Annex I parties before and after participation. *ω*(*i*,*j*) is calculated propensity scores of participating (*i*) and nonparticipating countries (*j*).

It is crucial to compare the actual and counterfactual outcomes to accurately estimate the program’s effect. This can prove to be a complex task in social science research, particularly because the same observations cannot be found before and after (with and without) a program, simultaneously. This study can establish the counterfactuals quantitatively and eliminate the bias using the differencing process [26]. The fixed regression equation model compares the observed changes in the levels of CO_2_ emissions and GDP for Annex I and non-Annex I parties. The coefficient of Δ*T*_*it*_ (i.e., ∅) from the program dummy variable, which is 1 if a country is affiliated to Annex I parties and captures the impact of the Protocol as follows:

$$\begin{array}{c} \left(Y_{it}-Y_{it-1})=\emptyset (T_{it}-T_{it-1})+\delta (X_{it}-X_{it-1})+(\eta_{i}-\eta_{i})+(\varepsilon_{it}-\varepsilon_{it-1}\right)\\ \Rightarrow \Delta Y_{it}=\emptyset \Delta T_{it}+\delta \Delta X_{it}+\Delta \varepsilon_{it} \end{array}$$ (2)

where *Y*_*it*_ is the environmental or economic performance indicator of country *i* in year *t*, *T*_*it*_ denotes the program impact, reflecting whether the country participates as an Annex I party. Other control variables are included in variable *X*_*it*_; *η*_*i*_ and *ε*_*it*_ indicate the unobserved time-invariant individual heterogeneity and other unobserved characteristics, respectively. Finally, Δ indicates the first difference. This analysis process controls not only for the time-varying covariates, but also for the unobserved time-invariant individual heterogeneity [26, 32].

### Model specifications

Two models are proposed to investigate the consequences of the Protocol. The environmental as well as the economic equations contain a program impact variable—an *Annex I* dummy—to capture the impacts of the Protocol as an Annex I party. This variable takes 1 if a country is an Annex I party and 0 otherwise.

To investigate the environmental impact of the Protocol, this study uses the logarithmic variables of *CO*_*2*_ *emissions* as the independent variable, as in the following equation:

$$\begin{array}{c} Ln({CO}_{2}\;Emissions)=\alpha_{0}+\alpha_{1}Annex\;1+\alpha_{2}Ln(GDP)+\alpha_{3}Oil+\alpha_{4}EUSE\\ +{\alpha_{5}ETS+\alpha }_{6}Recession+\varepsilon \end{array}$$ (3)

where *Ln*(*GDP*), the logarithmic variable of GDP, estimates the relationship between CO_2_ emissions and GDP. This study considers global and domestic energy, as well as commodity market volatility. The *Oil* variable is the national real oil price with regard to turbulence in the global energy market. The Recession dummy is added to reflect global market volatility, which is 1 if the sample participated in Annex I parties in the year of recession. This study regards 2008 as the year of recession, since the global financial crisis started from the summer of 2008. To reflect domestic energy policy, the *EUSE* and *ETS* dummy variables are included, which represent the amount of energy use and the status of participating in the emissions trading system (ETS), respectively. The *ETS* dummy variable takes 1 if a nation adopted the ETS in the year studied. Further, an error term *ε* is included.

Next, the economic impact model evaluates the impact of the Protocol on the GDP of each country:

$$\begin{array}{c} Ln(GDP)=\beta_{0}+\beta_{1}Annex\;1+\beta_{2}Ln(Capital)+\beta_{3}Ln(Labor)+\beta_{4}Ln(Human)\\ +\beta_{5}Oil+\beta_{5}EUSE+\beta_{6}ETS+\beta_{6}Recession+\epsilon \end{array}$$ (4)

This model is based on the Cobb–Douglas GDP production function which include the logarithmic variables of capital, labor, and human capital. Similar to the environmental impact model, this model includes the Protocol dummy for the program impact variable, *Oil* and *EUSE* variables, and *ETS* and *Recession* dummies to reflect global and domestic energy, as well as commodity market volatility.

These models are treated as a simultaneous equation system, namely, a two-stage least squares (2SLS) system, since the *GDP* variable in the CO_2_ equation is endogenous (i.e., correlated) if the second equation holds [32, 33]. The Hausman test for the first equation rejects the null hypothesis, that is, there is no correlation between GDP and the disturbance. *Ln*(*GDP*) is estimated by using the *Anne*x I dummy and other relevant variables, and then the first equation is estimated by using the fitted value of the second equation, $Ln\hat{(GD}P)$, as well as the *Ann*ex I dummy and other variables:

$$\begin{array}{c} Ln{(CO}_{2}\;\hat{Emi}ssions)=\gamma_{0}+\gamma_{1}Annex\;I+\gamma_{2}Ln\hat{(GD}P)+\gamma_{3}Oil+\gamma_{4}EUSE\\ +{\gamma_{5}ETS+\gamma }_{6}Recession+\varepsilon \end{array}$$ (5)

This procedure provides consistent estimates when the appropriate instruments are used [33]. The empirical models are estimated by using Stata.

### Selection of evaluation periods

The appropriate selection of the base and target year of the program is a crucial step in setting reliable counterfactual situations [26]. The base year is usually recognized as the year in which nations declare their commitment to the environment by officially signing a particular IEA. The Protocol has a relatively short history when compared with other IEAs. It was adopted in 1997 and was effective from 2005 onward. This study designates both the adoption year and the year of entry into force in order to observe the gap in the impact of the Protocol between the data adoption and entry into force.

Several scholars have used the target year for emission reductions as stipulated in the Protocol [19, 30, 34]. Previous literatures have estimated the impact of the Protocol by using data from before the end of the commitment period (e.g., analyses by Grunewald and Martínez-Zarzoso [4], Kim et al. [30], Kumazawa and Callaghan [8], and UNFCCC [5]). This study has set the target year as 2008 to focus on the impact of the Protocol before the commitment period. The target year was set as 2008, which is the start year of the first commitment period to secure a sufficient data for the impact evaluation.

## Data

To examine the environmental and economic effectiveness of the Kyoto Protocol, this study uses country-level panel data of 209 countries from 1997–2008. Based on this database, we conduct DID matching. Regarding the dependent variables of CO_2_ emissions and GDP, this study accepts data from the World Development Indicators (WDI) by the World Bank as reliable data [35], although various sources provide country-level data on CO_2_ emissions and GDP.

The empirical models contain the program impact variable to determine the effectiveness of the Protocol based on whether the parties belong to Annex I. Information on each country’s participation in the Protocol is extracted from the UNFCCC [10], the official site of the Secretariat of the Kyoto Protocol. The program effect variables of the models determine whether the parties belong to Annex I by allocating a value of 1 if the country is affiliated to Annex I in the focal year. For GDP function variables, this study uses gross fixed capital formation, the labor force participation rate (percentage of the population aged between 15 and 64 years), and adjusted savings–education expenditure (current United States Dollar (USD)). GDP and capital data are expressed in constant USD from 2000, while education expenditure is in current USD because of data limitations.

The *Oil* variables are based on annual data on the price of crude oil from the EIA [36], while the official exchange rate and consumer price index (CPI) of each country have been taken from the WDI [35]. We calculate the national real oil price with the price of crude oil, official exchange rate (Local Currency Unit (LCU) per USD, period average), and CPI, based on the following equation from previous studies: National real oil price for each country = [(Brent crude oil price (USD per barrel)) (Official exchange rate (LCU per USD, period average))] / (CPI (2010 = 100)) [37–39]. The *ETS* variable, which indicates the degree of domestic energy policy, is established based on the International Carbon Action Partnership (ICAP) Status Report 2015 [37, 39, 40]. This is also a dummy variable, which takes a value of 1, if we observe the status of adoption of the ETS in a certain year. The *EUSE* variable is from data on GDP per unit of energy used (constant at the 2011 purchasing power parity (PPP) in terms of $ per kg of oil equivalent) in the WDI [35]. Table 1 presents descriptive statistics of the relevant variables after the matching process. Using the matching process, the database is separated into two datasets, for 1997–2008 and 2005–2008.

**Table 1:** Descriptive statistics for the full sample after matching.

Variable	*N*	Mean	Std. Dev.	Min.	Max.
1997 base-year model					
*Annex I*	194	0.371	0.484	0	1
*Ln(CO*_*2*_ *emissions)*	194	10.225	2.169	4.733	15.556
*Ln(GDP)*	194	24.362	2.074	20.323	30.116
*Ln(Capital)*	194	22.810	2.172	17.111	28.373
*Ln(Labor)*	194	4.226	0.140	3.770	4.508
*Ln(Human capital)*	168	21.357	2.272	16.046	27.260
*Oil*	194	366.425	1793.574	0.100	18417.490
*EUSE*	174	8.395	3.991	1.200	22.759
*ETS*	194	0.144	0.352	0	1
*Recession*	194	0.469	0.500	0	1
2005 base-year model					
*Annex I*	113	0.673	0.471	0	1
*Ln(CO*_*2*_ *emissions)*	113	11.194	1.869	5.681	15.573
*Ln(GDP)*	113	25.388	1.888	20.367	30.116
*Ln(Capital)*	113	23.941	1.812	19.296	28.419
*Ln(Labor)*	113	4.245	0.104	3.892	4.450
*Ln(Human capital)*	113	22.835	1.823	17.804	27.260
*Oil*	106	123.100	983.976	.212	10073.03
*EUSE*	110	8.863	3.624	2.105	22.759
*ETS*	113	0.469	0.501	0	1
*Recession*	113	0.487	0.502	0	1

## Results

### Impact on CO_2_ emissions

In Table 2, the columns indicating environmental impact present the results of the impacts of Annex I parties on CO_2_ emissions. The R^2^ values are 0.870 for the 1997 base-year model and 0.749 for the 2005 base-year model.

**Table 2:** Estimated results of the matched DID model.

Model	Environmental impact		Economic impact	
Object variable	*Ln(CO*_*2*_ *emissions)*		*Ln(GDP)*	
Base year	1997	2005	1997	2005
Target year	2008	2008	2008	2008
*Annex I*	-0.618 (0.449)	-0.132 *** (0.038)	0.036 (0.155)	-0.070*** (0.015)
*Ln(Capital)*	-	-	0.701*** (0.058)	0.044 (0.032)
*Ln(Labor)*	-	-	0.107 (0.234)	-0.413 (0.317)
*Ln(Human capital)*	-	-	0.267*** (0.056)	0.186*** (0.034)
$Ln\hat{(GD}P)$	0.974*** (0.043)	0.810** (0.375)	-	-
*Oil*	0.000 (0.000)	-0.000 (0.000)	-0.000 (0.000)	-0.000 (0.000)
*EUSE*	0.022 (0.078)	-0.037** (0.015)	-0.007 (0.026)	0.006 (0.006)
*ETS*	0.638 (0.471)	0.027 (0.055)	-0.288* (0.163)	0.020 (0.025)
*Recession*	-0.151*** (0.206)	0.065 (0.062)	-0.172** (0.078)	0.100*** (0.015)
Constants	-13.632*** (1.201)	-9.028 (9.491)	2.423** (1.085)	21.793*** (1.349)
R^2^	0.870	0.749	0.985	0.917
Number of samples	149	99	149	99
Number of groups	81	52	81	52

***, **, and * denote the 1%, 5%, and 10% significance levels, respectively.

After eliminating the unmatched samples and missing values, 149 samples are used for the 1997 base-year model (two time pairs of 81 nations), and 99 samples are used for the 2005 base-year models (two time pairs of 52 nations).

First, the program impact variables—the Annex I dummies—show a negative sign in all models; however, only the coefficient of the 2005 base-year model exhibits statistical significance at the 1% level. These results indicate that, between 2005 and 2008, Annex I parties accomplished greater CO_2_ emission reductions than non-Annex I parties. These highly significant results suggest that it takes time for the Protocol to reduce the emissions of Annex I parties. It also indicates that imposing emission reductions targets has a beneficial impact on reducing CO_2_ emissions in the long run.

The above positive result of the 2005 base-year model is in accordance with previous discussions on the real impact of the Protocol [5], as well as previous studies that have showed that participating in IEAs has a positive impact on emission reductions [34, 41]. These works have shown that participating in international governance could efficiently mitigate and reduce pollution. This study can overcome the limitations of previous studies by controlling for both pre-program and post-program and treated-group and control group differences. Therefore, the estimated impacts of the Protocol have become more reliable and clearer.

Second, the GDP variables, namely, the instrumental variables, used to solve the problem of endogenous variables are positive and statistically significant for all models. The coefficients for the 1997 and 2005 base-year models are statistically significant, confirming that CO_2_ emissions increase with economic development. The empirical results imply that a 1% increase in GDP triggers an increase in CO_2_ emissions by around 1%. This result shows that economic growth is positively related with CO_2_ emissions. CO_2_ emissions are reported to increase along with economic development primarily because of the increase in the use of fossil fuel for industrial development [32–44]. As an extension of the previous studies, significant evidence is found herein to support a monotonic relationship between GDP and CO_2_ emissions.

This is in line with the result of the Recession dummy variable, which shows a significant and negative coefficient in the 1997 base-year model. Regarding the result of the Recession dummy, we expect an inverse relation in line with the result of the GDP variable because the Recession dummy reflects economic slowdown. In other words, there is a possibility that the economic depression may affect CO_2_ emission reductions.

Finally, the EUSE variable, the control variable shows significant coefficients in the 2005 base-year models. However, these results of the control variables are not only limited but also restricted, and thus further studies are needed to confirm the impact of those factors on CO_2_ emission reductions. The other control variables—national real oil price and status of participating in the ETS—show no statistically significant differences in CO_2_ emission reductions between the base and target years.

### Impact on GDP

The three columns from the left in Table 2 report the economic impacts of the Protocol. The R^2^ values (0.985 for the 1997 base-year model and 0.917 for the 2005 base-year model) demonstrate that all three models can explain more than 90% of the variation in GDP.

First, the Annex I dummy variable is highly significant with a negative sign only in the 2005 model. This finding shows that the negative impact of being an Annex I party on economic performance takes effect after having participated for a certain period. The economic performance of Annex I parties deteriorated by approximately 7% in 2005–2008. This means that Annex I parties, which are bound by reduction obligations, recorded lower economic growth than other comparable non-Annex I countries.

These findings concur with the empirical findings of Nordhaus and Boyer [9], namely, that an economic burden is placed on Annex I parties. These authors argue that, although most Annex I parties are developed countries, economic growth may be curtailed because of the socioeconomic costs, investments, and implementation of corresponding policies for emission reductions. Therefore, the economic outputs of Annex I parties are reduced because of the need to reduce energy, thus leading to an increase in production costs.

Besides the costs of emission reductions, this negative economic impact can be explained by theoretical evidence. Institutional factors, such as enforcement and implementation procedures or sanctions, can influence the impacts of the Protocol. Some scholars insist that the legalization and flexibility in the institutional mechanisms of IEAs can either improve or worsen their effectiveness [31, 45–47]. Legally binding environmental treaties or agreements are likely to have a positive impact on improving environmental performance. Further, flexibility mechanisms can facilitate rapid adjustments to new circumstances in the decision-making and implementation process.

Table 2 indicates that, while the coefficients of Capital in the 1997 base-year models and Human capital variables in all models are positive and statistically robust, those of Labor are not statistically significant in this analysis. With regard to the control variables for global and domestic energy and commodity market volatility, the Oil variable and EUSE dummy variables present no significant impact on the GDP of each country, similar to the environmental impact models. However, a negative impact of participating in the ETS and recession is observed in the 1997 base-year analysis. Hence, operating the ETS causes an economic burden. Moreover, economic performance in non-recession years is significantly higher than that of other countries from 1997 to 2008. The *Recession* dummy variable in the 2005 base-year model has positive signs with 1% significance level; however, it may take time to reflect the impact of recession in national economic performance.

### Predicted CO_2_ reductions and loss of GDP for Annex I parties

Table 3 provides the simulation results of predicting the impacts of the Protocol. These prediction values based on the 2005 base-year models, which have statistically significant Annex I dummy variables, are calculated for Annex I parties.

**Table 3:** Predictions of the impacts of the Kyoto protocol.

	Participating	Non-participating	(Non-participating)—(Participating)	%
CO_2_ (emissions, metric ton (MT))	21,224	24,219	2,995	14.111%
GDP (constant 2000 USD)	31,348	33,621	2,273	7.251%

The actual measured CO_2_ emissions were 26,423 MT in 2008 and 26,061 MT in 2007. The actual measured GDP was USD 35,327 billion in 2008 and USD 35,136 billion in 2007.

The values under the participating and non-participating columns help capture the differences between the actual and counterfactual situations. The CO_2_ emissions and GDP values under the participating column are estimated under the assumption that all countries participated as Annex I countries, based on real data for the 2005 base year. Annex I dummy variables take the value of 1. However, the non-participating column represents the assumption that the countries did not participate as Annex I countries. Thus, Annex I dummy variables take the value of 0.

Table 3 indicates that participating as an Annex I party produces a beneficial impact on CO_2_ emission reductions. The gap between participating and non-participating countries is 2,995 MT. Thus, if parties were not under the obligation to mitigate CO_2_ emissions as Annex I countries were, they would have emitted as much as 14% more CO_2_, as per the 2005 base-year model. In this respect, participating as Annex I parties with reductions obligations significantly affects CO_2_ emission reductions. In contrast, being an Annex I country has an adverse impact on economic growth. Approximately 7% of GDP growth—USD 2,273 billion—arises from the non-participating situation. This large gap indicates the considerable economic impacts of participating as Annex I parties.

Next, the economic loss and environmental benefit are directly compared using monetary measures. The total loss of GDP resulting from the participation in Annex 1 is as discussed above and is estimated to be USD 2,273 billion per year. For a direct comparison, the total CO_2_ reduction, estimated to be 2,995 MT per year, can be transformed into a monetary value. Tol conducted a meta-analysis of the marginal damage cost of carbon emissions based on the calculated values from 28 published studies. He found that the median and mean of the cost of damage are USD 14 and 93 per ton, respectively [48]. Using the median value, the total marginal benefit from mitigating CO_2_ emissions is approximately USD 42 billion, or approximately 2% of the total GDP loss. If the mean is used instead, the benefit increases to USD 279 billion or approximately 12% of the marginal damage to total GDP. This study applied the marginal damage cost of carbon emissions, which is effective for comparing costs and benefits of CO_2_ mitigation within a monetary value. However, this marginal cost does not reflect the entire damage due to CO_2_ emissions in the real world.

## Discussion

This study clarifies the impact of mitigating emissions and the economic burden on Annex I parties to the Protocol. The results indicate that although the impact of the Protocol on Annex I parties offsets economic growth; there are positive effects of emission reductions. The Protocol is unable to improve the performance of both the environmental and economic aspects. Moreover, in terms of the marginal benefit of the Protocol based on the marginal damage cost of carbon emission, the marginal benefit of emission reductions mitigates a limited portion of the GDP loss.

Since the adoption of the Kyoto Protocol in 1997, the international community has been paying close attention to its impact on both the environment and the economy [4–8, 10]. The annual Conference of Parties (COP), which began in 1995 for reviewing the implementation of the UNFCCC, provides a global roadmap for climate change action. Specifically, the 2015 United Nations Climate Change Conference (COP21) laid a strong foundation for a universal agreement on climate change by providing a new direction. The negotiations at COP21 concluded with the decision to embark on a new political course of action, requiring that all countries make an effort to limit the global temperature. This had a significant effect on the Paris Agreement in 2015 [10]. The international community is considering the effectiveness of the new agreement and addressing ways to enhance and implement each party’s Nationally Determined Contributions (NDCs) by 2020. For example, the international community agreed to pledge 100 billion USD to developing countries, to support mitigation and adaptation costs [49].

Even though, after COP21, the international community has discussed the rules, financial issues, and technical issues related to the implementation plans for combating climate change after the Paris Agreement enters into force in 2020, major obstacles still persist [50]. One of the major obstacles to the mitigation of global warming is the failure to reach a consensus in the climate deal. The US withdrew from the Paris Agreement, while China and India are revisiting their emission commitments [51–55]. For instance, discussions about the carbon market and emissions reduction have been postponed to COP26, to be held in 2021, since the non-cooperative countries, such as US, Russia, India, China, Brazil, and Saudi Arabia took the opposite stance [56]. These non-cooperative countries concern the political dilemma, which includes the cost effectiveness and trade-offs engendered by the Protocol. However, the absence of emission reductions and financial contributions from these countries threaten global climate governance in the future [54].

Since there have always been questions about the effectiveness of the Protocol in recent years, and the situation worldwide surrounding the global warming issue is still complex, the objectification of its effect may help understand the possibility of sustainable development. In this regard our results have policy implications for the Paris Agreement, a post-Kyoto regime for global climate change, which aims to limit global temperature rise to well below the 2°C target [10, 11]. The Paris Agreement adopted in December 2015 at COP 21 opens a new prospect in global climate efforts. This agreement is highly acclaimed, since it brings all parties into a common framework to mitigate CO_2_ emissions. First, in the Paris Agreement, each country has to set a goal to reduce emissions through the principle of nationally determined contributions (NDCs); however, no party has legally binding targets for emission reductions. The results indicate that participating as an Annex I party has a beneficial impact on reducing CO_2_ emissions. In other words, imposing a duty is an effective way of achieving UNFCCC’s global goal. Although the principle of NDCs that encourages all parties to improve their capacity to address climate change is a significant characteristic of the Paris Agreement, the principle of NDCs itself does not contain any obligations and sanctions. Only having more participants may not lead to greater effectiveness of the Protocol along with the United Nations Convention to Combat Desertification and the Basel Convention. In this respect, there is a concern that the Paris Agreement may remain an ineffective IEA [57].

Although a legally binding framework for emission reductions benefits the effective implementation of the Protocol, this finding shows a significant negative impact on economic performance among participants. This new course of the Paris Agreement for a sustainable low carbon future includes a requirement wherein both developed and developing countries report their emissions and implementation efforts in five-yearly cycles. To assess individual performance and further actions, global stocktaking will be conducted every five years. Scholars imply that one of the key challenges for the Paris Agreement is to secure multidimensional contributions including NDCs [11, 56, 58]. It is expected that low-income countries that have a low capacity to mitigate emissions may experience difficulties in adjusting to this new climate regime. There is a promising prospect for mitigating the economic burden in developing countries since the marginal cost of emission reduction is relatively lower than that in developed countries. The CDM may be a good example. Some developing countries achieve cost effective emission reductions through the CDM. Therefore, a well-defined global climate change framework, which balances the impacts on economic and environmental performance, could be more effective in those countries.

To secure the effectiveness of the Paris Agreement, the implications of this study mentioned above should be fully considered. To make a voluntary mechanism—NDCs in the Paris Agreement—effective, a systematic monitoring and evaluation mechanism must be established. A practical consideration for developing countries (e.g., a technical assistant) is also a crucial factor for improving the effectiveness of the agreement. The results show the significant negative impact of the Protocol on economic performance. The decline in GDP is thus a critical obstacle, especially for developing countries.

## Conclusions

This study investigated the impacts of the Kyoto Protocol on the Annex I parties’ CO_2_ emissions and GDP using country-level panel data for the periods 1997–2008 and 2005–2008. This study applied advanced statistical methods to systematically evaluate the effectiveness of the Kyoto Protocol. We combined the PSM and DID methods to consider the potential sample selection bias problem while examining the environmental and economic impacts of the Protocol. Through this approach, we could consider the differences in economic and environmental performance among countries that affect the impact of various IEAs in practice. Based on the challenges identified in this study, further research could adopt the impact evaluation method using broader data pertaining to various IEAs. The impact evaluation approach would provide a fundamental, but important understanding of the impact of IEAs.

The results suggest that the Kyoto Protocol has had significant positive impact on CO_2_ emission reductions and a negative impact on the GDP levels of Annex 1 Parties. The model’s predictions to capture the differences between the actual and counterfactual situations provide monetary value of economic loss and environmental benefit of participating in Annex I parties. Even though this study has grasped the overall effectiveness of the Kyoto Protocol with advanced methodologies, further consideration must be given to interpret this result.

First, although the matched DID approach was designed to minimize time-varying covariates and unobserved time-invariant individual heterogeneity, the result provides only the complete change among the Annex 1 countries. Therefore, we should not underestimate the situation in the Annex 1 countries, which consists of various countries. For example, a significant decline in emissions and economic performance from many of the former satellite states of the Soviet Union during the 1990’s had a significant impact on the overall effectiveness of the Protocol. Moreover, the empirical evidence in this study still shows the “trade-off” between reducing carbon emission and economic growth, however, the technological and structural changes in economy have led to increased energy efficiency and mitigated economic burden. This decoupling phenomenon between the CO_2_ emission and GDP is already shown in some countries like the U.S. and the U.K. In this regard, further research must consider this aspect and the role of the IEA to stimulate this phenomenon.

It is important that we introspect the meaning of sustainable “development” going forward. This study establishes the conceptual connection between the effectiveness of IEAs and sustainable development and showed the comprehensive understanding about the effectiveness of IEAs in the environmental and the economic performance by measuring GDP. However, we must be careful regarding the assumption that increase in the GDP is a desirable goal for sustainable development. There are limitations to and controversy regarding the use of GDP as a measurement of economic performance, even though GDP is still a very strong and readily available indicator of economic performance. Further analysis should consider the effectiveness of IEAs by diverging from the conventional limitations of measuring the economic performances to provide deeper understanding of sustainable development.

Before concluding the paper, limitations should be mentioned. First, there could be further improvement related to the marginal damage cost. Although the prediction based on the marginal damage cost of carbon emission revealed that the Protocol’s marginal benefit mitigates a limited portion of the GDP loss, there is no consensus among scholars regarding the marginal damage cost of carbon emissions [48, 59, 60]. Moreover, the damage cost does not adequately reflect the damage of emissions in the real society. Thus, additional efforts to quantify this damage cost are crucial for grasping the impact of the Protocol.

Second, the data set and study objectives can be improved to predict the marginal effect of the Protocol more accurately. Since this study focuses on the impact of the Kyoto Protocol before the start of the first commitment period, the periods analyzed were not long enough. The real consequences of international policies manifest only in the long term. Therefore, further research in this area could focus on the impact of other IEAs from a long-term perspective. In follow-up studies with longer observation periods, for example, analysis on the first (2008–2012) and second (2013–2020) commitment period are necessary to prove that IEAs for sustainable development have a positive impact on environmental and economic performance. Analyzing the database with longer time periods may provide ample clues about the decoupling phenomenon between CO_2_ emissions and GDP growth.

It should be noted that external shocks on energy demands need to be considered over the long term. For example, the COVID-19 pandemic has resulted in a temporary reduction in global CO_2_ emissions. Scholars insist that the pandemic has had a significant positive impact on the decline of global CO_2_ emissions, as domestic industrial activity and energy consumption have seen significant change during the crisis [61, 62]. Thus, a long term analysis on the effectiveness of an environmental regime should consider the impact of external shocks on factors affecting CO_2_ emissions and the economies of the participants.

Next, the level of stringency of the emission reduction target of the parties should be considered. Different emission reduction targets, burdens, and incentives among countries can have a significant impact on regime effectiveness, since the lenient target, in other word low incentive, lead to limited domestic efforts. Even though this study provided important empirical evidence on the overall effectiveness of the Protocol of Annex 1 countries, we did not consider the domestic differences in emission reduction target, burden, and incentive among parties. More analysis focusing on this aspect would be required to investigate the impact of the level of stringency of the emission reduction targets on the IEA effectiveness and to derive effective policy implications toward sustainable development.

Moreover, further research should consider the impact of the Protocol by taking into account the leakage of environmental damage among the non-Annex I parties. Since this study evaluates the overall effectiveness of the Kyoto Protocol among the Annex I countries, it is difficult to consider the consequences of the Protocol on the non-Annex I countries. Increasing emissions and economic growth in other parts of the world is also an important factor to consider in order to deepen our understanding of the effectiveness of the Protocol.

## List of acronyms

2SLS: Two-Stage Least Squares
CDM: Clean Development Mechanism
CER: Certified Emission Reductions
CO_2_: Carbon Dioxide
COP: Conference of Parties
CPI: Consumer Price Index
DID: Difference-in-Difference
EKC: Environmental Kuznets Curves
ETS: Emissions Trading System
EU: European Union
GDP: Gross Domestic Product
GHG: Greenhouse Gas
ICAP: International Carbon Action Partnership
IEA: International Environmental Agreement
IV: Instrumental Variables
LCU: Local Currency Unit
MT: Metric Ton
NDCs: Nationally Determined Contributions
ODA: Official Development Assistance
OECD: Organization for Economic Cooperation and Development
PSM: Propensity Score Matching
UNFCCC: United Nations Framework Convention on Climate Change
USD: United States Dollar
WDI: World Development Indicators
